# Long-Term Outcome After Implantation of the Ahmed Glaucoma Valve in Refractory Glaucoma

**DOI:** 10.7759/cureus.85230

**Published:** 2025-06-02

**Authors:** Styliani Alexia Papadonta, Kalliopi Kontopoulou, Elpida Kollia, Zaira Eleni Armeni, Reem Eliasson, Harald Schilling, Markus Kohlhaas, Sofia Fili

**Affiliations:** 1 Department of Ophthalmology, St. Johannes Hospital, Dortmund, DEU; 2 Department of Paediatric Ophthalmology, Moorfields Eye Hospital, London, GBR; 3 Department of Paediatric Ophthalmology, Manchester Royal Eye Hospital, Manchester, GBR; 4 Department of Ophthalmology, National and Kapodistrian University of Athens, Athens, GRC; 5 Department of Ophthalmology, Augenklinik Walsum, Duisburg, DEU

**Keywords:** ahmed glaucoma valve, conjunctival dehiscence, corneal decompensation, intraocular pressure, refractory glaucoma

## Abstract

Background

Glaucoma is the second leading cause of blindness, requiring effective and sustainable management through pharmacological, laser-assisted, or surgical therapy. The Ahmed Glaucoma Valve (AGV) (New World Medical, Inc., Rancho Cucamonga, CA) is a surgically implanted drainage system that can provide long-term reduction in intraocular pressure. This study retrospectively evaluates the outcomes of patients with complicated glaucoma, refractory to medical therapy, who had an AGV implanted at the Department of Ophthalmology of St. Johannes Hospital in Dortmund, Germany.

Material and methods

Preoperative and postoperative data of glaucoma patients who underwent an Ahmed valve implantation from January 2008 to December 2012 were obtained from hospital medical records over a maximum follow-up time of seven years. The evaluation included the indication for implantation, postoperative complications, and treatment success.

Results

The most common diagnosis was primary chronic open-angle glaucoma (42.8%), followed by secondary glaucoma (38.4%); 28.9% of patients were previously treated with trabeculectomy. The absolute success rate after one year was 51.6%, and the qualified success rate was 84.9%. A significant and sustained reduction of the intraocular pressure of 42.4%, from 28.9 ± 11.0 to 16.64 ± 7.2 mmHg, was observed after one year. The number of local medications required after the ocular surgery significantly decreased over time. The most common complication was ocular hypotony (30.8% at discharge).

Conclusion

The therapeutic success of AGV implantation in glaucoma patients in our clinic was very high. A significant and sustained reduction of intraocular pressure was achieved. One year postoperatively, complications occurred only sporadically.

## Introduction

In Western Europe, glaucoma is the second leading cause of blindness, following age-related macular degeneration [1]. In Germany, the prevalence of glaucoma among adults over the age of 40 is estimated to be between 3% and 5% [2]. Elevated intraocular pressure (IOP) is among the most significant risk factors [3]. Reducing IOP by 30-50% in patients with established glaucoma can halt disease progression and remains the only recognized preventive measure to date [4].

This reduction can be achieved through topical therapy with eye drops, systemic medication, laser procedures, or surgical interventions [1,5-7]. Each approach aims to achieve an individualized target IOP below which disease progression is not expected. At the time of this study (2008-2012), topical medication was the predominant first-line therapy. Although selective laser trabeculoplasty (SLT) has since gained recognition as a first-line option in the management of primary open-angle glaucoma (POAG), particularly following the results of the Laser in Glaucoma and Ocular Hypertension Trial (LiGHT), it was not yet widely adopted during the study period. Trabeculectomy remains the gold standard among surgical interventions and is typically reserved for cases in which pharmacological and less invasive treatments have proven insufficient [8]. Implanted glaucoma drainage devices, such as the Ahmed Glaucoma Valve (AGV) (New World Medical, Inc., Rancho Cucamonga, CA), which diverts aqueous humor to the subconjunctival space via a tube, have also demonstrated promising outcomes [9].

The AGV is one such drainage system [10]. It consists of a base plate made of medical-grade silicone, polypropylene, or porous polyethylene, a drainage tube, and a valve mechanism made of medical-grade silicone [11]. A unique feature of the AGV is its response to changes in IOP in both directions, responding to both elevated and low pressures, which significantly helps reduce the risk of hypotony [11]. Clinical studies have shown that the AGV achieves excellent outcomes in reducing and stabilizing IOP over the long term, with success rates comparable to those of trabeculectomy and the Baerveldt glaucoma implant [12-20]. Reported postoperative complications following AGV implantation include capsular fibrosis, erosion of the tube or plate edges, clinical failure, rarely infections, and hypotony [11]. A key factor supporting the use of the AGV is the significantly lower rate of vision-threatening complications associated with this implant compared to other surgical methods [21,22]. However, while the need for glaucoma medication is generally reduced after AGV implantation, it appears to remain higher compared to other implants. The aim of this study was to retrospectively evaluate the long-term outcomes and therapeutic success of patients with treatment-refractory glaucoma after implantation of an AGV (model FP7).

## Materials and methods

Patients who underwent AGV implantation (model FP7) for advanced, medically refractory glaucoma at the Department of Ophthalmology, St. Johannes Hospital in Dortmund, Germany, between January 2008 and December 2012 were included in this retrospective study. A total of 151 patients (68 women and 83 men) were enrolled in the study. In total, 159 surgeries were analyzed, as eight patients underwent bilateral implantation.

Inclusion criteria were as follows: a) medically refractory glaucoma, defined as IOP uncontrolled despite maximally tolerated medical therapy; b) history of failed glaucoma surgery or contraindication to trabeculectomy; and c) advanced glaucomatous optic neuropathy with corresponding visual field loss.

Exclusion criteria were as follows: a) active ocular neovascularization; b) phthisis bulbi or eyes with no light perception; and c) uncontrolled ocular inflammation or active uveitis.

Preoperative and postoperative data were extracted from patient records. Postoperative follow-up assessments were conducted at one to two weeks, four to eight weeks, three months, six months, one year, and annually thereafter, with a maximum follow-up period of seven years. These visits included assessment of IOP using Goldmann applanation tonometry, best-corrected visual acuity (BCVA), the number of antiglaucoma medications (AGMs), and slit-lamp examination.

Some key definitions used in this study are as follows:

Hypotony was defined as an IOP below 6 mmHg on two consecutive visits. When this condition persisted for more than three months postoperatively, it was classified as persistent hypotony.

Absolute success was defined as achieving and maintaining an IOP between 6 and 21 mmHg without the use of AGMs and without the need for additional glaucoma surgery. In contrast, qualified success referred to maintaining this IOP range with or without AGMs and/or following further glaucoma procedures.

Treatment failure was characterized by the occurrence of serious complications requiring implant replacement or explantation.

The study adhered to the principles outlined in the Declaration of Helsinki and received approval from a qualified clinical ethics consultant at St. Johannes Hospital.

Quantitative data are presented as mean ± standard deviation (SD). Preoperative and postoperative comparisons were analyzed using paired t-tests. A p-value <0.05 was considered statistically significant.

All AGV (model FP7) implantations were performed under general anesthesia by experienced glaucoma surgeons. A fornix-based conjunctival peritomy was created at the limbus, typically in the superior temporal quadrant, between two rectus muscles. Blunt dissection was used to develop a subconjunctival pocket by separating Tenon’s capsule. Hemostasis was achieved with bipolar cautery as needed. Mitomycin C (0.2 mg/mL) was applied beneath the conjunctiva using a soaked cotton pledget for three minutes to reduce postoperative fibrosis, followed by thorough irrigation with balanced salt solution. The AGV was primed with saline and fluorescein to verify valve function. The implant was positioned 8-10 mm posterior to the limbus and fixed to the sclera with 8-0 polypropylene sutures. The silicone tube was trimmed to the appropriate length, beveled anteriorly, and inserted through a 23-gauge needle tract into the anterior chamber. Care was taken to avoid contact with the iris or corneal endothelium, maintaining 2-3 mm of the tube within the anterior chamber. A bovine pericardium patch graft (Tutopatch®, Tutogen Medical GmbH, Neunkirchen am Brand, Germany) was placed over the exposed tube to prevent erosion. Finally, the conjunctiva was repositioned and closed using a running 10-0 nylon suture to ensure a watertight seal.

## Results

The patient cohort had a mean age of 69.7 ± 16.4 years, with 45% of the patients being female. The AGV was implanted in 91 right eyes (57.2%) and 68 left eyes (42.8%).

Based on diagnostic classification, the most common diagnosis at the time of admission was POAG, which accounted for 42.76% of all treated eyes. Secondary glaucomas, including uveitic, neovascular, aphakic, post-traumatic, pigmentary, and angle-closure subtypes, comprised 38.4% of cases. Pseudoexfoliation glaucoma was observed in approximately 10% of patients, and for the purposes of this study, was categorized separately due to its distinct clinical profile.

A substantial proportion of patients had previously undergone multiple ocular surgeries. The most frequent prior intervention was trabeculectomy (28.9%), followed by pars plana vitrectomy (22.0%) and transscleral cyclophotocoagulation (TSCPC) (15.7%).

**Figure 1:**
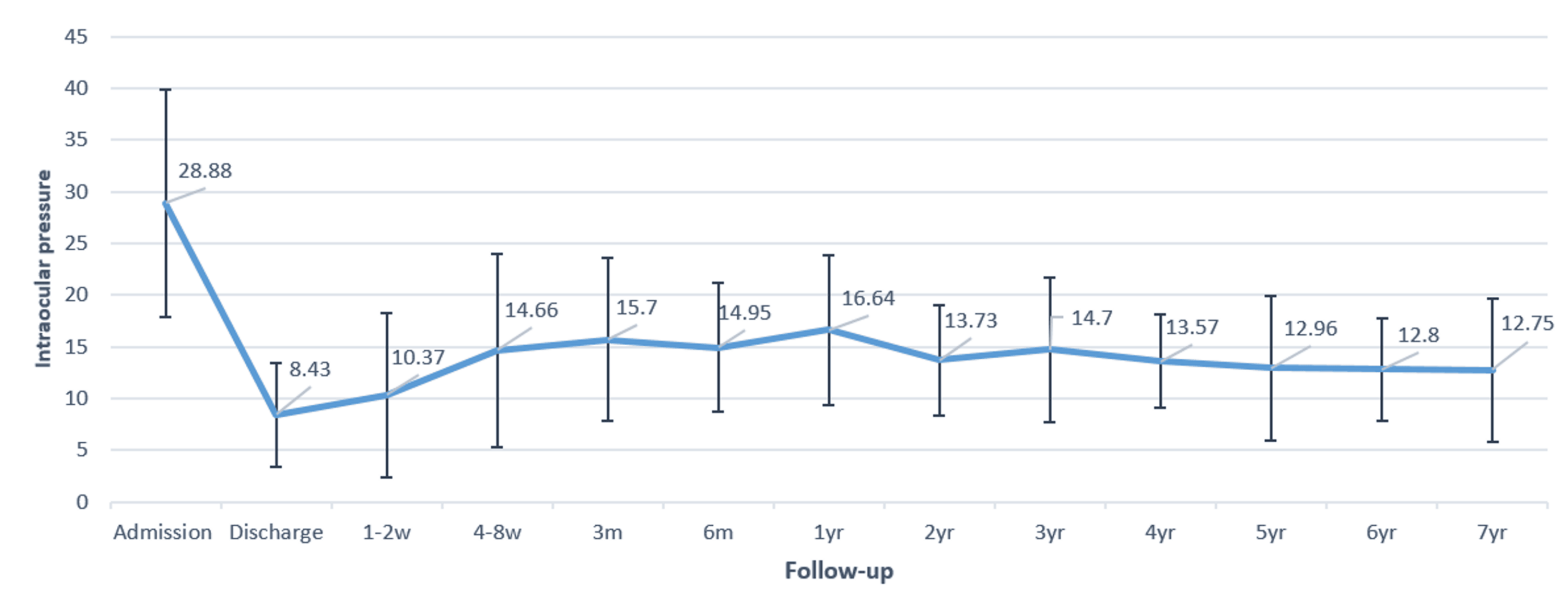
Graph showing the trend of mean intraocular pressure (IOP) (in mmHg) at admission, at discharge, and at follow-up visits for up to seven years

The mean preoperative IOP was 28.9 ± 11.0 mmHg. At two weeks postoperatively, the mean IOP was reduced to 10.37 ± 7.94 mmHg, and at three months, it was 15.7 ± 7.86 mmHg. From the third postoperative month onward, the IOP reduction was statistically significant (p < 0.05). Twelve months after surgery, the mean IOP remained significantly reduced compared to the preoperative value (16.64 ± 7.23 mmHg). The maximum mean reduction of 55.9% from baseline was observed at seven years postoperatively (Figure 1).

**Figure 2:**
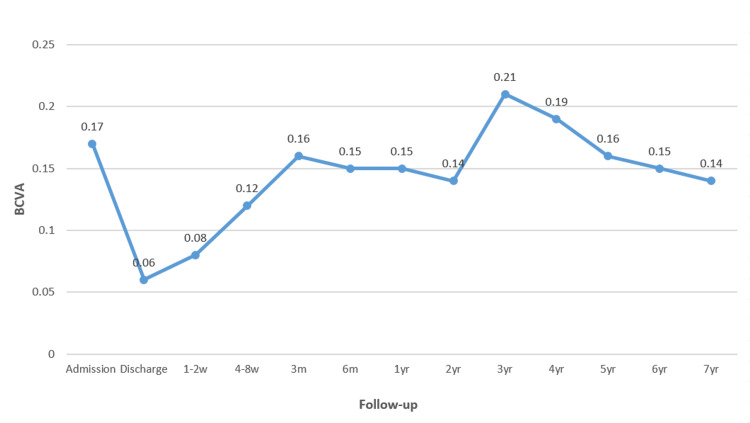
Graph showing the trend of the mean best corrected visual acuity (BCVA) (in Snellen decimal) at admission, at discharge, and at follow-up visits for up to seven years

BCVA did not show a statistically significant difference between baseline and long-term follow-up. A temporary decline in visual acuity was noted during early postoperative visits, but gradual improvement was observed over time. However, only partial restoration of preoperative visual acuity was achieved by the end of the seven-year follow-up period (Figure 2).

**Figure 3:**
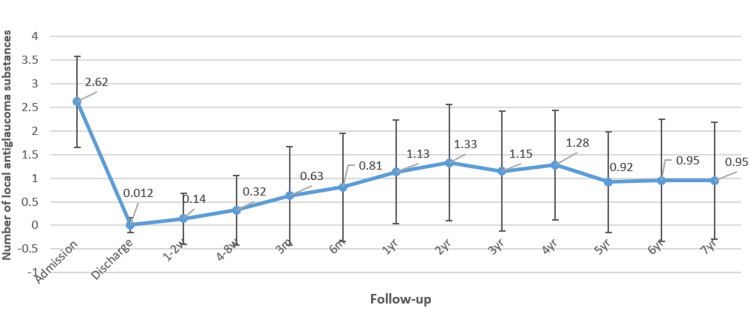
Graph of the number of local antiglaucoma substances at admission, at discharge, and at follow-up visits for up to seven years

The number of topical AGMs significantly decreased from a preoperative average of 2.6 to between 0.8 and 1.0. However, a gradual increase in the number of required medications was observed beginning one to two weeks after surgery, continuing over the subsequent four years. Following this period, the number of medications stabilized at approximately 0.96 ± 1.24, representing a 63% reduction from baseline at the seven-year follow-up (Figure 3).

During the early postoperative phase, a transient elevation of IOP, commonly referred to as the hypertensive phase, was observed. This phenomenon typically occurs within one to eight weeks following AGV implantation and is considered a normal postoperative response related to wound healing and bleb maturation, rather than a surgical complication [22,23]. In our cohort, elevated IOP (>21 mmHg) was recorded in approximately 3.33% of eyes at one to two weeks postoperatively, increasing to 12.17% at four to eight weeks, which is consistent with previously reported rates.

The most common complication was transient ocular hypotony (IOP < 6 mmHg), occurring in 30.8% of cases at discharge. Although early hypotony was frequent, most episodes were transient and resolved without surgical intervention or significant vision loss. Choroidal detachment was the next most frequent complication, observed in 7.6% of eyes. The incidence of hypotony declined steadily over time, dropping to 2.67% at 12 months. Using our definition of persistent hypotony as IOP < 6 mmHg on two consecutive visits, no cases persisted beyond 12 months, although isolated new hypotony episodes were noted between years two and seven. Importantly, no cases of phthisis bulbi or endophthalmitis were reported. A detailed timeline of complications is provided in Table 1.

**Table 1:** Postoperative complications

Follow-up	Ocular hypotony, n (%)	Corneal decompensation and/or endothelial touch, n (%)	Choroidal detachment, n (%)	Conjunctival dehiscence, n (%)	Macular edema, n (%)
Discharge	49 (30.8)	1 (0.6)	12 (7.6)	0	0
1-2 weeks	26 (21.7)	1 (0.8)	14 (11.7)	5 (4.2)	0
4-8 weeks	13 (11.3)	0	11 (9.6)	2 (1.7)	1 (0.9)
3 months	6 (5.7)	1 (0.9)	1 (0.9)	1 (0.9)	2 (1.9)
6 months	4 (4.8)	1 (1.2)	2 (2.4)	2 (2.3)	3 (3.6)
1 year	2 (2.7)	5 (6.7)	1 (1.3)	1 (1.3)	3 (4.0)
2 years	2 (3.8)	2 (3.8)	0	0	3 (5.7)
3 years	3 (7.5)	4 (10.0)	0	0	0
4 years	0	2 (7.1)	0	0	1 (3.6)
5 years	2 (7.1)	2 (7.1)	0	0	1 (3.6)
6 years	1 (4.8)	1 (4.8)	0	0	0
7 years	1 (4.2)	1 (4.2)	0	0	0

In 33 of the 159 cases (20.75%), additional surgical interventions were necessary. AGV explantation was performed in 11 cases (6.92%), and valve replacement in two cases (1.26%). Reasons for explantation included conjunctival dehiscence (n = 2, within six months), valve exposure (n = 5), pressure decompensation (n = 1), hypotony with choroidal detachment and corneal decompensation (n = 1), hypotony with endothelial tube touch (n = 1), and corneal decompensation with elevated IOP (n = 1). Valve replacement was required within four to eight weeks postoperatively due to pressure decompensation in one case and valve exposure in the other.

TSCPC was reserved for a select subset of patients (eight cases, 5%) who exhibited persistently elevated IOP following AGV, primarily in eyes with limited visual potential. Additionally, AGV revision surgery was performed in three cases (1.9%). These revisions occurred within three months postoperatively - two cases due to elevated IOP alone and one due to elevated IOP with encapsulation. Of the total 24 secondary procedures, 16 took place within the first 12 months following AGV implantation (Table 2). 

**Table 2:** Indications for follow-up surgeries DMEK, Descemet membrane endothelial keratoplasty; TSCPC, transscleral cyclophotocoagulation

Surgeries follow-up	Ahmed valve revision, n	Ahmed valve explantation, n	TSCPC, n	Replacement of Ahmed valve, n	Ahmed valve tube shortening without DMEK, n	DMEK plus Ahmed valve tube shortening, n	Penetrating keratoplasty, n
Discharge	-	-	-	-	1	-	-
1-2 weeks	-	-	-	-	1	-	-
4-8 weeks	-	-	-	2	-	-	-
3 months	2	-	2	-	-	-	-
6 months	1	5	1	-	-	-	-
1 year	-	3	-	-	-	-	1
2 years	-	1	1	-	-	1	1
3 years	-	2	1	-	1	1	-
4 years	-	-	-	-	-	1	-
5 years	-	-	-	-	1	-	-
6 years	-	-	-	-	-	-	-
7 years	-	-	3	-	-	-	-

Nine additional surgeries were performed due to endothelial tube touch or corneal decompensation. These included tube shortening in four cases, Descemet membrane endothelial keratoplasty in three cases, and penetrating keratoplasty in two cases.

## Discussion

This study aimed to evaluate the clinical outcomes of patients with therapy-refractory glaucoma following implantation of the AGV (model FP7). The most frequent diagnosis at baseline was POAG (42.8%), followed by secondary glaucomas (38.4%) and pseudoexfoliation glaucoma (10%). Notably, 28.9% of patients had undergone prior trabeculectomy, underscoring the refractory nature of the cohort.

At one year, absolute success - defined as IOP between 6 and 21 mmHg without additional medications - was achieved in 51.6% of eyes. Qualified success, allowing for the use of AGMs, was attained in 84.9%. These outcomes are comparable to prior literature. For instance, Budenz et al. reported a one-year cumulative failure rate of 16.4%, increasing to 44.7% at five years, with failure most commonly due to uncontrolled IOP [23,24]. Similarly, Christakis et al. observed a cumulative failure probability of 51% at three years [22], while Riva et al. reported a success rate of 63.1% after three years [1].

In the present study, a robust and stable reduction in IOP of approximately 50% was achieved. This is consistent with the findings of Christakis et al. [22], who reported an average IOP reduction of 49% (to 15.7 ± 4.8 mmHg) in patients with AGV implants after a three-year follow-up. After five years, the average IOP was 15.8 ± 5.2 mmHg [25]. Budenz et al. [23] found an average postoperative IOP of 15.4 ± 5.5 mmHg after one year and 14.7 ± 4.4 mmHg after five years. Elbaklish et al. [26] reported an average IOP of 16.6 ± 3.4 mmHg after one year.

The observed reduction in medication usage in this study aligns with the literature: Christakis et al. [22] noted a 42% decrease in medication usage to an average of 1.8 ± 1.4 medications, while Budenz et al. [24] reported a requirement for 1.8 ± 1.3 AGMs after one year and 2.2 ± 1.4 after five years. In contrast, Elbaklish et al. [26] reported a significantly higher requirement of 3.9 ± 0.3 medications one year post-AGV implantation. In our cohort, the early postoperative increase in medication use was likely due to a prophylactic approach aimed at minimizing IOP spikes during the immediate postoperative period. This strategy may have contributed to better short-term pressure control and a lower incidence of the hypertensive phase. In certain cases, particularly in eyes with advanced disease or limited visual potential, AGMs were maintained long-term due to cautious tapering practices or concerns about subclinical pressure fluctuations. The retrospective nature of the study and the lack of standardized bleb imaging may also have influenced these individualized treatment decisions.

A transient hypertensive phase, defined as an IOP elevation occurring one to eight weeks postoperatively, was observed in approximately 12-16% of eyes. This phenomenon is well-documented following AGV implantation and may relate to early fibrotic encapsulation around the valve plate. It is important to note that the hypertensive phase is not a surgical complication per se, but rather a postoperative pressure response. In our study, this was typically self-limiting or managed with temporary medication adjustments. The relatively low incidence compared to the literature may reflect early AGM initiation, patient selection (e.g., low aqueous producers, post-vitrectomy eyes), or the flow-regulating properties of the AGV.

Transient ocular hypotony (IOP < 6 mmHg) was the most commonly observed early postoperative complication, occurring in 30.8% of eyes at discharge. Although frequent initially, the incidence of hypotony declined steadily over time, reaching 2.67% at 12 months. Persistent hypotony was defined as an IOP < 6 mmHg on at least two consecutive follow-up visits. Notably, no cases of persistent hypotony were observed beyond the 12-month follow-up, although isolated late-onset episodes were documented between years 2 and 7.

Several factors may have contributed to the relatively high early hypotony rate observed, including tight scleral wound closure, potentially leading to over-filtration, reduced aqueous humor production due to advanced disease stage, prior TSCPC in 15.7% of eyes, potentially impairing ciliary body function, and absence of pre-insertion flow testing of AGVs.

Despite the high early incidence, the majority of hypotony episodes were transient, resolved spontaneously, and did not result in significant vision loss. Cases with persistent hypotony lasting beyond three months or those requiring surgical revision were classified as treatment failures.

Choroidal detachment was the next most frequent complication (7.6%). Peritubular leaks were typically managed conservatively. No cases of phthisis bulbi or endophthalmitis were observed.

Christakis et al. reported that 52% of patients experienced complications within the first three years post-surgery [25]. Similarly, Elbaklish et al. reported complications in 47% of patients [26]. In the present study, complications occurred in 39% of patients at discharge, but only in 17.5% of patients after three years, indicating a lower complication rate compared to previous studies.

Follow-up surgeries were required in 15.1% of patients in the present study. Common indications included valve exposure, conjunctival dehiscence, and hypotony-related issues. In comparison, Christakis et al. reported a higher rate of 38% within the first three postoperative years [25]. Budenz et al. also observed a higher reoperation rate of 20.8% within the first five years following surgery [24].

Due to the retrospective nature of this study, bleb morphology was not consistently documented, which represents a recognized limitation. The lack of standardized imaging likely influenced postoperative management decisions, including the timing of AGM tapering. Future prospective studies should incorporate uniform bleb grading and imaging protocols to better assess bleb characteristics and their impact on outcomes.

Additional limitations include the heterogeneous patient population, variability in treatment regimens, and differences in surgical technique among surgeons, all of which may have influenced the results.

## Conclusions

AGV implantation demonstrated favorable outcomes in this real-world cohort with therapy-refractory glaucoma. Although early postoperative hypotony was frequent, most episodes were transient and resolved without intervention. Long-term IOP control and complication rates were comparable with existing literature. Our findings support AGV as an effective option for managing complex glaucoma, while underscoring the need for prospective studies with standardized imaging and follow-up protocols.
